# Evolving health policy and regulatory oversight of medicinal cannabis in Australia: lessons for sustainable integration

**DOI:** 10.1186/s42238-026-00394-z

**Published:** 2026-01-27

**Authors:** Enoch Chi Ngai Lim, Chi Eung Danforn Lim

**Affiliations:** 1 Translational Research Department, Specialist Medical Services Group, Earlwood, NSW Australia; 2 NICM Health Research Institute, Western Sydney University, Westmead, NSW Australia; 3 School of Life Sciences, University of Technology Sydney, Ultimo, NSW Australia

**Keywords:** Medical cannabis, Australia, Regulation, Access

## Abstract

Since federal legalisation in November 2016, Australia’s medical cannabis regulation has evolved into a complex framework that involves the Therapeutic Goods Administration, state health departments, and multiple professional oversight bodies. This narrative review examines the policy hurdles and coordination gaps that have emerged between 2016 and 2024, providing a longitudinal perspective on the ongoing debate. Evidence shows patient access expanded dramatically: the number of prescriptions jumped from 231 in 2017 to more than one million by early 2024, generating an estimated market value of AUD $445.6 million. Yet notable barriers remain, including monthly costs that average between $200 and $600, uneven availability in remote areas, and variable knowledge levels among general practitioners and specialists. Core obstacles, therefore, include the tangled federal-state regulatory maze, persistent equity problems for rural patients, challenges in embedding medical cannabis into standard clinical pathways, and a supply chain that still relies heavily on imported products. A comparative analysis with Canada’s opt-out insurance model, Germany’s pharmacist-led dispensing, and the Netherlands’ community-growth scheme highlights Australia’s relatively restrictive approach, particularly in terms of reimbursement and comprehensive provider training. Policy options thus centre on extending Pharmaceutical Benefits Scheme (PBS) subsidies, building domestic cultivation and manufacturing capacity, mandating uniform educational modules for prescribers, and streamlining inter-agency oversight. This review aims to inform Australian reforms while also offering transferable lessons for other jurisdictions contemplating or revising therapeutic cannabis programmes.

## Introduction

During the past decade, the global landscape for medical cannabis has shifted rapidly; more than fifty nations now allow patients to use cannabis-based therapies under controlled conditions [1]. This trend marks a significant policy shift, moving away from blanket prohibition towards decisions grounded in clinical evidence and patient needs. Australia’s own path in this arena illustrates the gains and obstacles that accompany such change, providing lessons for regulators and public health officials worldwide.

At the federal level, Australia legalised medical cannabis in November 2016 by amending the Narcotic Drugs Act of 1967; the country thus became the first to achieve nationwide reform through legislation rather than court rulings or popular referenda [2]. This milestone laid the groundwork for a multilayered regulatory system that engages several government agencies and state jurisdictions, thereby broadening patient access while complicating the timely and uniform implementation. Though grounded in domestic concerns, the Australian case study resonates internationally, shaping and being shaped by global best practice in medical cannabis policy. Sitting between jurisdictions that either severely limit or overwhelmingly liberalise access, the country’s model privileges patient safety through clinical governance while cautiously broadening availability channels [3].

To chart Australia’s path, this review examines the policy and regulatory obstacles encountered between 2016 and 2024, an era marked by shifting laws, evolving public opinion, and evolving scientific evidence. Its aims are fourfold: to untangle the layers of regulation, to measure the fairness and reach of access, to report on integration into routine care, and to highlight reform openings revealed by external comparison. Coverage extends from federal Therapeutic Goods Administration (TGA) rules through state licences, clinician viewpoints, lived patient stories, and the upstream supply-chain realities that bind them together. Australia’s journey speaks directly to other jurisdictions considering medical cannabis, showcasing both innovative regulatory advances and lingering structural challenges. A close examination of these successes and setbacks yields the evidence needed for sound, implementable policies that genuinely fit within the broader healthcare system.

## Methodology

This narrative review draws on published accounts of interviews with patients and healthcare professionals, as reported in peer-reviewed studies and parliamentary submissions, as well as quantitative analysis of regulatory figures and official policy texts. Its analytical lens reviews on health policy theory, regulatory science, and health services research to provide a comprehensive understanding of how medical cannabis has established itself in Australia. Quantitative data were obtained from TGA dashboard reports, the Authorised Prescriber registry, transcripts of parliamentary inquiries, and searches of PubMed and Cochrane. Position statements by clinical bodies and submissions from patient groups provided additional context. For comparative insight, the team reviewed government publications and peer-reviewed studies from Canada, Germany, the Netherlands, and Israel. Coverage spans from November 2016, when Australian federal law first opened the door, to December 2024, capturing the entire story of rollout and refinement. It weighs policy outputs more than clinical outcomes, probing how regulatory rules, implementation hitches, and funding flows have converged, or clashed, in everyday healthcare. Literature was included if it examined Australian regulatory developments, utilisation trends, or relevant international policy features. Comparator jurisdictions were selected based on maturity of national programmes and relevance to Australia’s prescriber-led regulatory design. As a narrative review, limitations include reliance on administrative datasets subject to revision, survey response bias, and an evolving regulatory environment.

## Background and literature review

### Evolution of Australian medical cannabis policy

Australia’s medicinal cannabis framework has evolved through successive implementation cycles, moving from cautious early safeguards toward operational streamlining as utilisation expanded. Key reforms can be interpreted as responses to implementation pressures identified in earlier phases rather than isolated policy shifts. Australia’s medical cannabis policy was developed in response to advocacy sparked by well-publicised cases of children with epilepsy whose families sought cannabinoid therapies. The 2015 death of Dan Haslam, a long-time campaigner for such treatment, strengthened public and parliamentary momentum for reform [4]. His story, along with others, prompted federal inquiries that culminated in legislative amendments in 2016. The Special Access Scheme (SAS) and Authorised Prescriber (approval to prescribe specified unapproved medicines) pathways predate cannabis reforms but were extended in 2016 to include cannabis products. These pathways have since been refined, including the introduction of a category-based system in 2021 and further streamlining in 2024 [5]. Early on, each application had to name a particular product, so doctors faced a patchwork of paperwork while suppliers dealt with fragile stock lines. To simplify the process, a November 2021 overhaul replaced product names with a five-category system based on cannabinoid ratios rather than specific products, slashing median turnaround from weeks to just 1–3 business days [6]. The evolution of Australia’s medical cannabis policy can be mapped across several key regulatory milestones. Table 1 summarises major national and state developments from 2016 to 2025 and indicates whether each change primarily expanded access, constrained access, or had mixed effects.

**Table 1:** Evolution of australia’s medicinal cannabis regulatory milestones (2016–2025)

Year	Regulatory Development	Key Feature/Outcome	Jurisdiction/Agency	Access Direction
2016	Legalisation via *Narcotic Drugs Act 1967* amendment	Establishment of national legal access pathways	Federal – TGA/ODC	Expanded
2017–2019	Early SAS & AP framework implementation	Product-specific approvals; limited prescriber uptake	Federal/States	Mixed
2020	Queensland removes additional state approval	Streamlined prescriber access	Queensland Health	Expanded
2021	Five-category product system introduced	Simplified classification; shorter approval times	TGA	Expanded
2023	GMP (TGO 93) standards mandated	Improved quality; temporary supply disruptions	TGA/ODC	Mixed
2024	SAS/AP Online system launched	Paperless submissions; improved transparency	TGA	Expanded
2025	AHPRA Rapid Regulatory Response Oversight Group established	Proactive monitoring of high-volume prescribers	AHPRA/Medical Board of Australia	Restricted

*TGA *Therapeutic Goods Administration, *ODC *Office of Drug Control, *SAS *Special Access Scheme, *AP *Authorised Prescriber, *GMP *Good Manufacturing Practice, *TGO *Therapeutic Goods Order, *AHPRA *Australian Health Practitioner Regulation Agency, *MBA *Medical Board of Australia, *Qld *Queensland

### Policy rationale for categorisation

The 2021 shift to a category-based approval framework arose from accumulated implementation challenges. Product-specific approvals became increasingly inefficient as product availability fluctuated and substitutions were required, generating repeat applications and administrative delays for clinicians and regulators alike. Reframing approvals around cannabinoid composition rather than individual products addressed these operational constraints while preserving clinical oversight, reflecting iterative regulatory learning driven by real-world system pressures [6].

To support national regulation, Australian states pursued distinctly local strategies. Queensland, for example, eliminated its own approval step in June 2020; however, Tasmania retained its rule that requires prescribers to hold Tasmanian registration [7]. As a result, access varies markedly between jurisdictions, creating geographic disparities that reflect the federal system of shared responsibilities rather than a cannabis-specific issue [8]. Looking to 2024, several process improvements have been introduced: applications now move almost entirely online through the SAS/AP portal, agencies share data more smoothly, and high-volume prescribing faces sharper review [2]. Meanwhile, the Office of Drug Control has expedited licensing while maintaining core security measures, and the Australian Health Practitioner Regulation Agency (AHPRA) continues to provide clear guidance on telehealth prescriptions [9].

### International comparisons

Canada’s framework offers the broadest benchmark because the Cannabis Act of 2018 created a single, federal rule set that also lowers barriers for doctors who prescribe [10]. The system now serves a large national medical cannabis patient population, thanks to clear access routes and competitive prices from regulated growers [11]. Germany introduced its own scheme in 2017 and has since linked prescriptions directly to insurance, cutting patients’ monthly out-of-pocket costs to around €130–170, a sharp contrast to Australia’s AUD $400–500 [12]. Germany’s statutory medical cannabis framework ensures pharmaceutical-grade quality through mandatory Good Manufacturing Practice (GMP) standards and strict regulatory oversight, supporting safe and consistent patient access [13]. The Netherlands operates the world’s most controlled system through government-managed cultivation and pharmaceutical-grade standardisation, serving approximately 25,000 patients with consistent product quality but limited access expansion [14]. Israel leads in clinical research integration with over 110 active clinical trials and 112,000 patients, demonstrating the potential for evidence-based programme development [15]. To contextualise Australia’s regulatory design, Table 2 compares major international frameworks in terms of access routes, insurance coverage, and prescriber training.

**Table 2:** International models of medical cannabis regulation and funding

Country	Regulatory Model	Insurance/Public Funding	Average Monthly Cost (local currency)	Prescriber Requirements
Australia	Federal SAS/AP; state licensing overlays	No PBS coverage (except Epidiolex^®^)	AUD $200–600	Limited training; optional CPD
Canada	*Cannabis Act 2018* – federal licensing	Private insurance partial coverage	CAD $150–200	Standardised online authorization
Germany	Statutory insurance-linked scheme (2017)	Public insurers cover approved indications	€130–170	Mandatory clinical justification
Netherlands	Government monopoly cultivation model	Limited public subsidy	€200–250	Physician authorization only
Israel	National medical programme with research integration	Partial subsidy	ILS 300–400	Structured training for certified clinicians

*PBS* Pharmaceutical Benefits Scheme, *CPD *Continuing Professional Development, *AUD *Australian Dollar, *CAD *Canadian Dollar, *€ *Euro, *ILS *Israeli Shekel

Australia’s regulatory model reflects selective policy transfer rather than wholesale adoption of international approaches. While pharmaceutical-grade manufacturing and prescriber-led access mirror elements of European frameworks, Australia deliberately avoided insurance integration and mandatory prescriber certification seen in some jurisdictions. Compared with Canada’s broader access environment, Australia maintained greater regulatory conservatism, shaped by its legislative-first reform pathway and reliance on unapproved product access routes.

### Research gaps in the Australian context

Recent systematic reviews confirm gaps in evidence, particularly around chronic pain, anxiety, and long-term outcomes [16, 17]. The Cannabis as Medicine Survey (CAMS) series provides the most comprehensive Australian data, tracking user patterns from less than 1% prescribed usage in 2016 to 73% of surveyed cannabis users reported prescribed access by 2022 [18]. The clinical research infrastructure has expanded through the $4 million NHMRC-funded Australian Centre for Cannabinoid Clinical and Research Excellence (ACRE) and the Lambert Initiative for Cannabinoid Therapeutics [19, 20]. However, methodological challenges include regulatory barriers for research ethics approval, product standardisation issues, and difficulties maintaining study blinding [20]. Economic analyses remain limited, with international studies showing variable cost-effectiveness ratios, ranging from cost-saving to over US$451,800 per quality-adjusted life year, depending on the condition and setting [16]. Australian-specific health economic evaluations are particularly needed for consideration under the Pharmaceutical Benefits Scheme (PBS). From a policy perspective, priority research gaps include high-quality trials for high-volume indications such as chronic pain and anxiety, standardised product comparability to enable pooled analysis, and Australian health-economic evaluations aligned with PBS decision frameworks. Addressing these gaps is central to future reimbursement feasibility.

## Medical cannabis usage statistics and trends in Australia

### Historical prescription data 2017–2024

Australia’s medical cannabis utilisation has demonstrated an extraordinary growth trajectory since programme inception. The progression from 231 regulatory applications in 2017 to over one million total prescriptions by 2024 represents a 4,329-fold increase, making medical cannabis one of the fastest-growing therapeutic areas in Australian healthcare [21]. Annual prescription growth patterns reveal several distinct phases. The period from 2017 to 2019 showed exponential percentage increases (1,011% and 882%, respectively) from a small base, reflecting the initial programme implementation and growing awareness [22]. The 2019–2021 period demonstrated sustained high growth rates (129% and 112%), indicating programme maturation and broader clinical adoption [23]. Applications decreased in 2022 (117,000 approvals) during the transition period. Numbers rebounded in 2023 with 120% growth compared with 2022 [24]. Current utilisation data for 2024–2025 shows sustained momentum with over 33,520 SAS approvals in the first two months of 2025, representing a 47% increase compared to 2024 [24]. This growth trajectory positions Australia as one of the world’s largest medical cannabis markets, based on patient numbers and prescription volume.

### Drivers of growth

This extraordinary expansion reflects a convergence of structural and behavioural factors rather than a sudden emergence of new clinical indications. Consumer survey data indicate that many early patients had already been using cannabis illicitly for therapeutic purposes and subsequently transitioned into the regulated system as prescriber pathways became more accessible [3, 18, 25]. At the same time, the emergence of specialised prescribing clinics, increasing clinician familiarity, and iterative regulatory streamlining lowered practical barriers for new patients. Growth therefore reflects both formalisation of pre-existing use and incremental expansion into new patient populations.

### Patient demographics and medical conditions

Chronic pain dominates medical cannabis prescriptions, comprising 59% of all TGA approvals as of 2022 data [26]. This reflects both the limited evidence base for cannabis in pain management and the substantial unmet clinical need in chronic pain populations. Anxiety disorders represent the second-largest indication at 23% of approvals, followed by sleep disorders (4%), cancer-related symptoms (4%), and insomnia (3%) [26]. Patient demographic analysis from the CAMS-22 survey (*n* = 3,323) reveals a relatively balanced gender distribution, with 53% male and 47% female users, challenging assumptions about gender-based usage patterns [25]. The average patient age of 46.4 years indicates medical cannabis adoption across middle-aged populations rather than concentrated in elderly or young adult demographics [25]. Educational and employment patterns among prescribed users show higher rates of tertiary education and employment compared to illicit users, suggesting prescribed access may be influenced by socioeconomic factors [25]. This finding raises concerns about equity due to differential access based on educational attainment and economic resources. The CAMS-18 survey provides additional condition breakdown showing pain conditions (36.4%), mental health conditions (32.8%), sleep disorders (9.2%), neurological conditions (5.2%), and cancer-related conditions (3.8%) [3]. These patterns mirror international experience, but Australia’s regulatory settings influence the mix of patient conditions.

### Authorised prescriber numbers and geographic distribution

Healthcare provider engagement has grown substantially, but it remains concentrated in specific geographic areas. Authorised prescriber numbers increased from 144 doctors in 2017 to 3,088 nationwide by January 2025, with 911 prescribers submitting applications in February 2025 alone [26]. This represents approximately 5% of Australia’s registered medical practitioners, indicating a continued opportunity for expansion of professional engagement. SAS prescriber data shows 5,284 active prescribers by 2022, comprising 4,374 SAS-B (case-by-case access to unapproved medicines) prescribers and 910 SAS-A (limited defined access circumstances) prescribers, with an additional 1,246 Authorised Prescribers [22]. New South Wales leads in prescription volume, followed by Victoria and Queensland, reflecting both population distribution and state-specific policy implementations [27]. Geographic disparities remain pronounced, with rural and remote areas facing significant access challenges despite the expansion of telehealth [28]. Tasmania demonstrates the impact of restrictive state policies, which require both prescribers and dispensers to maintain Tasmanian registration, resulting in lower approval numbers relative to the population [27].

### Product types and cannabinoid preferences

Dried flower products represent the largest market segment, accounting for 39–42% of prescriptions, which reflects patient preferences for inhaled delivery and potentially cost considerations [25]. Oral solutions and oils comprise 33–36% of prescriptions, traditionally the predominant formulation in early programme years [26]. Pastilles and tablets exhibit rapid growth, accounting for 14% of prescriptions, indicating a shift toward conventional pharmaceutical formats [24]. The TGA’s five-category classification system, implemented in November 2021, provides detailed insight into cannabinoid preferences. THC-dominant products (categories 4–5) comprise 52% of prescriptions [21], reflecting their established therapeutic use in conditions such as chronic pain and chemotherapy-related nausea. While psychoactive effects may occur, these are generally regarded as secondary pharmacological effects rather than a therapeutic requirement, and clinical practice commonly aims to minimise psychoactivity where possible. Balanced THC: CBD products (category 3) and CBD-dominant products (categories 1–2) each represent 17% of prescriptions, suggesting diverse patient needs and clinical applications [26].

### Cost analysis and economic impact

The patient cost burden remains substantial, with monthly expenses ranging from AUD $200 to $600 for typical treatment regimens [29]. Regional variations show that Adelaide and Canberra are at the lower end (AUD $200–220 monthly), while Brisbane, Sydney, and Tasmania cluster at AUD $280–300 monthly [30]. Reported cost ranges reflect variation in product type, dosing, consultation frequency, and dispensing fees, and represent approximate monthly patient out-of-pocket expenditure. These costs represent a significant financial burden, with 47.6% of patients reporting costs place “significant strain” on their finances [3]. The absence of Pharmaceutical Benefits Scheme coverage creates particular hardship, as patients bear the full treatment costs, unlike most prescription medicines. The sole exception, Epidiolex^®^ for Dravet syndrome epilepsy, demonstrates the impact of PBS listing in reducing costs from $24,000 annually to $31.60 per prescription, the general patient PBS co-payment in 2025 [31]. Market valuation reflects rapid growth, with the sector reaching AUD $445.6 million in revenue for 2024-25, projected to grow at a 33.6% compound annual growth rate through 2030 [32]. Conservative estimates suggest the market could reach AUD $4.53 billion by 2029, indicating substantial economic significance beyond current levels [27].

## Key policy and regulatory challenges

### Federal–State division of regulatory authority

Australia operates a federal constitutional system in which responsibility for healthcare regulation is shared between the Commonwealth and the states. The Commonwealth regulates therapeutic goods, manufacturing, and importation through national agencies such as the TGA and Office of Drug Control, while states retain authority over poisons scheduling, prescribing permits, and jurisdiction-specific professional practice conditions. Consequently, state-level variation in medicinal cannabis access reflects established legislative arrangements rather than cannabis-specific inconsistency. This division explains why Queensland could remove additional state approvals while Tasmania retained more restrictive prescriber requirements.

### Regulatory framework complexity

Australia’s regulatory system resembles other Schedule 8 (controlled medicines requiring additional safeguards) medicines but adds extra complexity through cannabis-specific GMP evidence, multiple product categories, and varied state permit requirements. The system involves federal agencies (TGA for therapeutic goods regulation, ODC for controlled substance licensing, AHPRA for professional oversight), state health departments for practitioner permits, and multiple professional regulatory bodies [33]. Federal-state jurisdiction coordination presents ongoing challenges with inconsistent implementation approaches across states and territories. Queensland’s removal of state approval requirements in June 2020 contrasts sharply with Tasmania’s maintenance of prescriber restrictions, creating what patients describe as a “postcode lottery” [8]. These variations require patients to navigate different regulatory requirements based on their geographic location rather than their clinical needs. The TGA approval process, although streamlined through 2021 reforms, retains complexity due to categorical requirements and clinical justification standards. SAS-B applications require a detailed clinical rationale, including treatment history, conventional therapy failures, and safety considerations [34]. While online applications have reduced processing time to 1–3 business days, the initial application burden continues to deter participation by healthcare providers [35]. Quality control requirements under TGO 93 (mandatory quality standard for medicinal cannabis) demonstrate regulatory complexity through Good Manufacturing Practice standards for imports and domestic production. The implementation of mandatory GMP requirements from July 2023 led to supply chain disruptions, as products not meeting standards were withdrawn from the Australian market [36]. Country-specific evidence requirements for GMP compliance add administrative burden for international suppliers while ensuring product quality [37].

### Recent regulatory developments and Multi-Agency coordination

AHPRA and the Medical Board of Australia initiatives reflect growing concerns about high-volume prescribing practices and professional standards. The identification of eight practitioners who prescribed over 10,000 scripts within six months, including one nurse practitioner who prescribed up to 31,000 scripts, prompted enhanced oversight measures [38]. High prescribing volume alone does not establish inappropriate practice. Regulatory concern instead focuses on whether high-throughput models maintain adequate patient assessment, documentation, follow-up, and management of conflicts of interest, particularly within telehealth or vertically integrated clinic settings. AHPRA’s response, therefore, emphasises patient safety and professional standards rather than numerical thresholds alone. Joint regulator forums in February 2024 addressed concerns about medicinal cannabis prescribing and established information-sharing protocols [39]. The TGA’s regulatory evolution includes the transition to paperless applications through the SAS/AP Online System, effective from July 2024, which streamlines administrative processes while maintaining clinical oversight [2]. Category-based approvals, which replace product-specific applications, represent the most significant regulatory simplification, reducing approval complexity while maintaining therapeutic equivalence standards [6]. Inter-agency coordination challenges persist despite the improvement in communication protocols. Current legal barriers prevent the comprehensive sharing of TGA-AHPRA data, limiting visibility into prescribing patterns and potential regulatory concerns. Consultation processes for information-sharing legislative provisions indicate a recognition of coordination gaps that require structural reform. Operational regulatory challenges include supply chain volatility, which requires product substitution capabilities, varying state Schedule 8 permit requirements, and inconsistent real-time prescription monitoring system implementations [40]. These challenges highlight the need for enhanced regulatory harmonisation across jurisdictional boundaries.

AHPRA has also strengthened its regulatory oversight through its Regulating New and Changing Healthcare work, which includes the establishment of a Rapid Regulatory Response Oversight Group [41]. This group meets quarterly to identify emerging risks among regulated practitioners, including in high-demand medicine areas such as medicinal cannabis accessed via telehealth. Under this framework, AHPRA monitors prescribing and dispensing patterns, issues guidance to practitioners, and takes action against outlier behaviour. For example, in mid-2025, AHPRA issued new guidance reminding prescribers that medicinal cannabis is mostly Schedule 8, and that prescribing must meet professional and safety obligations (including proper patient assessment, medical records, and avoiding inappropriate first-line use) [42]. These measures show a shift: not just reacting to complaints, but proactively identifying and managing risks. They respond to concerns about patient safety in settings where clinics offer streamlined access, telehealth prescribing, or where conflicts of interest may arise.

### Access and equity issues

PBS decisions are based on evidence, cost-effectiveness, and opportunity costs compared with other therapies. A key structural constraint is that most medical cannabis products are accessed as unapproved medicines via SAS/AP pathways, whereas PBS listing typically requires defined indications, robust comparative evidence, and cost-effectiveness analyses. This creates a policy mismatch in which widespread clinical use can expand without triggering the evidentiary thresholds required for public reimbursement. High costs partly reflect manufacturer pricing. Value-based pricing strategies could help align costs with therapeutic benefit. Cost barriers represent the most significant impediment to access, with patients bearing the full treatment costs in the absence of Pharmaceutical Benefits Scheme coverage. Monthly treatment costs of AUD $400–500 average create particular hardship for fixed-income patients, elderly populations, and those with chronic conditions requiring ongoing therapy [43]. Geographic disparities in access demonstrate systematic inequities between urban and rural populations. Rural patients face combined challenges of limited local prescriber availability, higher travel costs for consultations, and limitations in pharmacy access [44]. While telehealth has reduced some geographic barriers, the underlying cost burden remains unchanged regardless of delivery modality. Patient eligibility criteria, although theoretically flexible through physician discretion, create practical barriers due to clinical justification requirements and conventional therapy failure documentation [45]. The 18-week average wait time between the initial consultation and receiving the first doses reflects systemic access challenges that extend beyond regulatory approval timeframes [3]. Healthcare provider qualification challenges compound access issues due to limited education on the endocannabinoid system in medical curricula and insufficient clinical guidance for prescribing decisions [46]. Professional uncertainty about dosing, drug interactions, and monitoring protocols creates reluctance to prescribe, particularly among general practitioners serving rural populations [35]. Several systemic factors continue to constrain affordability and equitable distribution. Table 3 consolidates the principal economic and regulatory barriers to medical cannabis access in Australia and aligns each barrier with corresponding policy responses.

**Table 3:** Economic and access barriers in the Australian medicinal cannabis market

Barrier Type	Description	Impact on Patients	Policy or Regulatory Response	Feasibility
Cost	Monthly out-of-pocket AUD $400–600	Financial hardship; discontinuation of therapy	PBS listing of evidence-based indications; value-based pricing	Medium
Geographic inequity	Rural shortages of prescribers and pharmacies	Delayed care; travel burden	Expand telehealth; harmonise state rules	High
Regulatory complexity	Multi-agency requirements (TGA, AHPRA, State Health)	Administrative burden; provider reluctance	Unified federal-state portal for approvals	Medium
Prescriber knowledge gap	< 10% of clinicians with formal training	Under-prescribing; variable quality of care	National curriculum and continuing education module	High
Supply chain reliance	98% imported products	Vulnerability to disruption and price fluctuation	Domestic cultivation and GMP incentives	Low-Medium

*AUD *Australian Dollar, *PBS *Pharmaceutical Benefits Scheme, *TGA *Therapeutic Goods Administration, *AHPRA *Australian Health Practitioner Regulation Agency, *GMP *Good Manufacturing Practice

### Patient Knowledge, misconceptions and educational challenges

Patient education challenges reflect both historical cannabis stigma and limited evidence-based information resources. The CAMS survey findings that 32% of users were unaware they could access medical cannabis legally, and 47.8% didn’t know a willing prescriber, indicate substantial patient education gaps [3]. Misconceptions about medical cannabis regulation, product quality, and therapeutic applications persist among patient populations. Product composition knowledge gaps affect 25.8% of users who report not knowing their cannabis composition, creating safety concerns and suboptimal therapeutic outcomes [25]. Quality consistency concerns affect 23.9% of patients, who report significant batch-to-batch variation, thereby undermining treatment reliability [3]. Healthcare provider education limitations contribute to patient knowledge gaps through inconsistent clinical information and varying professional attitudes toward medical cannabis. Only 6% of healthcare providers received formal medical cannabis training during professional education, while 60% obtained information through workshops and conferences of variable quality [34]. Consumer protection mechanisms remain underdeveloped compared to those for conventional pharmaceuticals, with limited adverse event reporting systems and insufficient post-market surveillance, creating information gaps about real-world safety and effectiveness [35].

### Clinical integration challenges

Healthcare provider confidence levels remain problematic, with 52% of general practitioners feeling uncomfortable discussing medical cannabis with patients [34]. Over two-thirds of GPs report inadequate knowledge about medical cannabis, while only 54% of psychiatrists and 57% of GPs support prescription availability [35]. Integration with existing medical treatments presents complex challenges through limited drug interaction data and unclear monitoring protocols. Healthcare providers express uncertainty about safe dosing regimens and administration routes, particularly when used as adjuvant therapy with conventional medications [3]. The off-label use of most medical cannabis products creates additional clinical uncertainty in the absence of formal clinical practice guidelines [47]. Patient monitoring and follow-up protocols remain poorly standardised across healthcare providers and clinical settings. Adverse event reporting relies heavily on voluntary systems rather than systematic pharmacovigilance, creating potential safety gaps [25]. Common side effects, including dry mouth (61.5%), increased appetite (59.2%), and drowsiness (54.7%), require clinical management strategies that many providers lack training to implement [3]. Workplace implications affect 9.3% of patients due to concerns about drug testing, necessitating clinical consideration of employment impacts in treatment planning [25]. Driving impairment concerns with THC-containing products create additional clinical management challenges requiring specialised knowledge about impairment timelines and detection methods [3].

### Supply chain and quality control

Australia’s dependence on international imports creates supply chain vulnerabilities, with approximately 98% of medical cannabis products imported rather than domestically produced [46]. Import data shows heavy reliance on Canadian suppliers (73% of 7,587 kg imported in 2021), creating exposure to international regulatory changes and supply disruptions [23]. The implementation of quality control through TGO 93 standards demonstrates both regulatory success and industry challenges. Mandatory GMP requirements effective July 2023 improved product quality but created market disruptions as non-compliant products were withdrawn [36]. The requirement for country-specific GMP evidence adds complexity for international suppliers while ensuring pharmaceutical-grade standards [37]. Manufacturing licence requirements for domestic production involve complex security assessments and quality management systems aligned with pharmaceutical standards [23]. Implementing cost recovery through industry fee structures creates additional barriers for smaller manufacturers while ensuring regulatory sustainability [23]. Product standardisation challenges affect both imported and domestic products, primarily due to limited cannabinoid profiling requirements and insufficient testing for contaminants. Meeting supply requirements at licensed facilities improves quality assurance but adds regulatory complexity and a cost burden, ultimately affecting final product pricing [23].

## Case studies and examples

### Implementation challenges across States in Australia

Queensland’s removal of its extra approval step in 2020 improved access. Yet, this expansion also drew criticism for its limited oversight and risks associated with vertical integration. This change allowed any registered practitioner to prescribe medical cannabis without state-specific approvals, reducing administrative burden and improving patient access [48]. Prescription volumes in Queensland increased substantially following this reform, indicating the impact of regulatory streamlining on access outcomes. Tasmania’s restrictive system, which requires local registration, remains a barrier, particularly for telehealth prescribing. The requirement for both prescribers and dispensers to maintain Tasmanian registration creates particular challenges for telehealth services and reduces available provider options [27]. Patient advocacy groups report cases of Tasmanian residents travelling interstate for consultations or relocating to access treatment, highlighting the impact of state-level regulatory variations. New South Wales demonstrates ongoing policy evolution through parliamentary inquiries and regulatory reviews. The 2024 cannabis legalisation inquiry reflects growing political attention to access barriers, while the scheduled Drug Summit indicates comprehensive policy reconsideration [49]. In NSW, Schedule 8 cannabis prescriptions may be electronic, computer-generated and signed, or handwritten, consistent with NSW Health guidelines [50].

### Patient access experiences

Rural patient experiences reveal systematic access challenges despite the availability of telehealth services. Case studies from patient advocacy groups describe patients in remote areas facing combined barriers of limited internet connectivity for telehealth consultations, no local pharmacy willing to stock medical cannabis products, and prohibitive travel costs for in-person appointments [44]. Financial hardship cases demonstrate the impact of cost barriers on treatment access and adherence. Patient advocacy submissions describe cases of elderly pensioners unable to afford ongoing treatment, parents reducing their own medication doses to provide for children with epilepsy, and cancer patients foregoing therapy due to cost [51]. These cases highlight the contrast between Australia’s universal healthcare principles and the reality of medical cannabis access. Successful patient stories typically involve urban residents with higher incomes accessing specialised clinics and consistent product supply. These positive experiences emphasise the importance of educated prescribers, reliable supply chains, and patient education in achieving therapeutic outcomes. However, these successful cases remain concentrated among socioeconomically advantaged populations and urban locations.

### Medical business integration models

Specific medical cannabis clinics represent a successful model for integrating medical businesses, providing dedicated expertise and streamlined access pathways. Specialised clinics have emerged, some promoting structured patient pathways and education. However, peer-reviewed assessments highlight both benefits and risks, including inconsistent standards and business model pressures [28, 35]. These models achieve higher patient satisfaction and clinical outcomes through specialised knowledge and dedicated resources. However, the specialised clinic model presents potential patient safety risks through business model pressures and limited oversight mechanisms. Commercial incentives may influence prescribing patterns, as clinics that depend on patient volume for revenue may potentially lead to inappropriate prescribing or inadequate assessment of conventional treatment options [35]. The concentration of high-volume prescribing in specialist clinics has attracted regulatory scrutiny, particularly regarding standardisation of clinical protocols and professional oversight [38]. Additionally, patient continuity of care may be compromised when specialist clinics operate independently from established healthcare systems, potentially creating fragmented medical records and communication gaps with primary healthcare providers [3].

Telehealth integration models demonstrate successful reduction of geographic barriers while highlighting remaining cost and supply chain challenges. CannaAid Medical and similar providers have developed standardised telehealth protocols enabling rural access while maintaining clinical standards [30]. However, these models remain limited by underlying cost barriers and the availability of pharmacies. The telehealth delivery model introduces specific patient safety concerns related to the limitations of remote assessment and reduced clinical oversight. Physical examination constraints in telehealth consultations may compromise comprehensive patient assessment, particularly for complex conditions requiring detailed neurological or musculoskeletal evaluation [38]. The absence of in-person monitoring creates challenges for detecting adverse effects, drug interactions, or changes in patient condition that may require treatment modification [35]. Telehealth also makes it easier for patients to switch between providers, sometimes referred to as “doctor shopping.” When histories are poorly shared, that can open the door to polypharmacy or inappropriate dosing [25].

Integrated care models that weave medical cannabis into broader treatment pathways show encouraging early results, yet their reach is still narrow. Pain clinics that add cannabis to a multimodal strategy have reported fewer opioid prescriptions and noticeable gains in patient satisfaction. However, the approach tends to flourish only in large cities that can attract the needed expertise and resources. Although these integrated teams enhance continuity and communication, they also expose patients to safety risks stemming from knowledge gaps and institutional barriers. Many medical providers in traditional clinics lack knowledge of medical cannabis, leaving them unsure how to dose, monitor, or identify potential adverse effects related to it [35]. Worries about liability, along with facility rules that vary widely, often discourage providers from incorporating cannabis into their usual treatment regimen. This results in patchy healthcare delivery, leaving patients uncertain about what to expect [46]. Scarcity of centres with an integrated model of care means that people in rural or regional areas may resort to loosely organised alternatives, exposing them to the hazards of fragmented treatment and incomplete information [44].

## Discussion

### Synthesis of findings

Australia’s rollout of medical cannabis reveals ongoing tensions between the objectives of patient access and the obligations of regulatory safety, as shown in Table 4. The system has recorded impressive increases in both patient numbers and prescribers, all while upholding product quality and clinical oversight. Persistent bottlenecks in access, however, signal that policy implementation remains partial and that structural obstacles are far from resolved. The coexistence of rapid growth and persistent access barriers reflects uneven distribution of regulatory burden within the system. While reforms such as category-based approvals and online submissions reduced friction for specialised providers and experienced prescribers, many general practitioners, particularly in regional settings, continue to face deterrents including limited training, uncertainty around monitoring and dosing, and residual administrative complexity. Growth has therefore been concentrated within specialised clinics and telehealth models rather than diffused evenly across the healthcare system.

**Table 4:** Systemic challenges and policy levers for sustainable integration

Challenge	Underlying Issue	Short-Term Action	Medium- to Long-Term Strategy
Fragmented regulatory oversight	Inconsistent TGA–AHPRA–state coordination	Establish data-sharing agreements	Legislative harmonisation of federal–state roles
High treatment costs	Lack of PBS coverage and domestic production	Interim co-payment assistance	PBS subsidy for proven indications; local manufacture
Professional knowledge gap	Limited medical curriculum content	Mandatory CPD module for prescribers	Integration of endocannabinoid science into medical education
Patient safety concerns	Rapid telehealth expansion and variable oversight	AHPRA Rapid Regulatory Response monitoring	Development of national clinical guidelines and audit systems
Supply volatility	Import dependency and price instability	Encourage short-term stockpiles	Invest in domestic GMP-certified production facilities

*TGA *Therapeutic Goods Administration, *AHPRA *Australian Health Practitioner Regulation Agency, *PBS *Pharmaceutical Benefits Scheme, *CPD *Continuing Professional Development, *GMP *Good Manufacturing Practice

High cost remains the leading barrier to equitable access, with Australians shouldering some of the steepest out-of-pocket expenses found anywhere. This financial load disproportionately burdens older patients, people in remote regions, and those with chronic diseases who need long-term treatment. Absent inclusion in the Pharmaceutical Benefits Scheme, affordable access for these vulnerable groups hinges on a targeted policy remedy. The difficulties in engaging health providers mirror long-standing problems in medical training, concerns about legal liability, and uneven access to robust clinical evidence. That most cannabis prescriptions now come from specialised clinics shows that these centres have built a viable business model. Yet, it also highlights the system’s failure to weave cannabis care into everyday health practice. While this narrow focus may enhance patient outcomes in those settings, it simultaneously limits access due to high costs and the uneven distribution of such clinics across regions.

### Policy implications

In the short term, policy efforts should focus on enhancing regulatory coordination by increasing information sharing among agencies and aligning state-level implementation practices. The ongoing review of the TGA-AHPRA data-sharing rules is therefore a critical building block for more reliable oversight and quality assurance. In the medium term, attention should shift to lowering cost barriers by listing rigorously proven indications on the Pharmaceutical Benefits Scheme. Australia’s adoption of the German model of integrating new therapies into insurance schemes could achieve this aim without sacrificing cost-effectiveness or clinical scrutiny. Over the longer run, fostering local production will help cut reliance on imports and support competitive pricing. Canada’s framework for licensed growers demonstrates how to maintain quality standards while fostering market competition, thereby ensuring both supply security and affordable prices. To realise these reforms, education for health providers must be embedded in university curricula and supported by continuous learning opportunities. Regulatory boards should collaborate to develop standard training modules and clear practice guidelines that address knowledge gaps and clarify the boundaries of professional liability.

### Lessons for other jurisdictions

Australia’s medical-cannabis programme provides helpful lessons for places planning similar systems. Its shift from approving single products to granting category-wide licences shows how regulators can learn and adapt over time. At the same time, the Australian case highlights the drawbacks of a convoluted, multi-level framework and reminds future planners that any access scheme must keep costs at the forefront. Australia’s experience highlights several features that other jurisdictions may wish to emulate, including the transition to category-based approvals, the implementation of a unified online submission system, and the enforcement of pharmaceutical-grade quality standards, all of which demonstrate how regulatory access can expand without compromising patient safety. At the same time, the Australian case underscores important pitfalls to avoid: persistent reliance on out-of-pocket patient funding, fragmented federal–state regulatory requirements, and non-standardised prescriber education have collectively constrained equity and impeded integration into routine care. Jurisdictions contemplating medical cannabis reform may therefore benefit from addressing reimbursement architecture, regulatory harmonisation, and clinician training early in policy design, rather than relying on incremental, post-hoc corrections once utilisation has already expanded.

### Future directions

In 2025, the TGA initiated a formal inquiry into the use of unapproved medical cannabis products accessed through the Special Access Scheme. This review highlights concerns about the rapid expansion of prescribing, the adequacy of clinical oversight, and the role of commercial interests [52]. Priorities for future research include trials that measure the clinical effectiveness of cannabinoid therapies in Australian populations, health economic evaluations that inform PBS listing, and detailed studies on how to implement the best care delivery models in routine practice. On the policy front, growing calls to legalise recreational cannabis create an urgent need for a coherent framework that aligns medical and non-medical use, supports stronger international partnerships on quality standards, and anticipates the health-system shifts required by a larger patient base. Technology initiatives under consideration include upgrades to the electronic prescribing system, dedicated telehealth platforms, and seamless integration of remote patient-monitoring tools. These initiatives promise to overcome geographic access barriers, strengthen clinical oversight, and deliver more consistent, higher-quality patient experiences and outcomes.

## Conclusion

Australia’s medical cannabis rollout between 2016 and 2024 demonstrates both the country’s progress and the work that remains to be done before the policy fully integrates into everyday healthcare. The leap from 231 applications in 2017 to over one million prescriptions in early 2024 underlines a pressing unmet medical need and confirms that the changing regulatory framework can drive adoption when patients, prescribers, and manufacturers understand it. Yet persistent hurdles—high prices, remote clinics that lack stock, and sparse training for prescribers—keep implementation from being uniform or fair. Core problems include tangled rules that vary from state to state, unequal access for rural and low-income populations, different clinical pathways that prescribers find vague due to limited guidance, and a fragile supply chain that still relies on overseas imports and suffers from inconsistent quality checks. To move forward, policy should widen the PBS listing to evidence-based indications, strengthen local cultivation and manufacture, standardise education for all prescribers, harmonise federal and state rules, and solidify the infrastructure that supports clinical trials. Research must then assess the real-world benefits and costs, test delivery models that work in diverse settings, and prioritise patients’ safety.

## Data Availability

No new data generated from this review. All data analysed during this study are included in this published article.

## References

[1] Hassoun A, Radhakrishnan M, Lamsdorf AJ, et al. Global regulatory landscape for medical cannabis: A systematic review. Cannabis Cannabinoid Res. 2021;6(5):389–98. 10.1089/can.2020.0017.33998863

[2] Therapeutic Goods Administration. Medicinal cannabis guidance documents, Australian Government Department of Health. 2024. Available from: https://www.tga.gov.au/products/unapproved-therapeutic-goods/medicinal-cannabis-hub.

[3] Lintzeris N, Mills L, Dunlop A, et al. Medical cannabis use in the Australian community following introduction of legal access: the 2018–2019 online Cross-Sectional cannabis as medicine survey (CAMS-18). Harm Reduct J. 2020;17:37. 10.1186/s12954-020-00377-0. 32513180 10.1186/s12954-020-00377-0 PMC7278204

[4] Medicinal cannabis campaigner Dan Haslam dies after battle with cancer. ABC News. Available from: https://www.abc.net.au/news/2015-02-25/medicinal-cannabis-campaigner-dan-haslam-dies/6260522.

[5] Australian Government Department of Health. Special access scheme. Therapeutic Goods Administration. 2024. Available from: https://www.tga.gov.au/accessing-medicinal-cannabis-patient.

[6] Therapeutic Goods Administration. Changes to the Special Access Scheme and Authorised Prescriber Scheme for medicinal cannabis products. November 2021. Available from: https://www.tga.gov.au/products/unapproved-therapeutic-goods/medicinal-cannabis-hub.

[7] Queensland Health. Medicinal cannabis products. 2024. Available from: https://www.health.qld.gov.au/public-health/topics/medicinal-cannabis/regulation/products.

[8] Senate Community Affairs References Committee. Current barriers to patient access to medicinal cannabis in Australia. Commonwealth of Australia. 2020. Available from: https://www.aph.gov.au/Parliamentary_Business/Committees/Senate/Community_Affairs/MedicinalCannabis.

[9] Australian Health Practitioner Regulation Agency. Joint regulator forum on medicinal cannabis prescribing. February 2024. Available from: https://www.ahpra.gov.au/News/2024-02-20-medical-cannabis-treatment.aspx.

[10] Government of Canada. Cannabis Act (S.C. 2018, c. 16). 2018. Available from: https://laws-lois.justice.gc.ca/eng/acts/C-24.5/.

[11] Government of Canada. Cannabis Act (S.C. 2018, c. 16). 2018. Available from: https://laws-lois.justice.gc.ca/eng/acts/C-24.5/.

[12] Häußermann A, Wetzel D, Knopf H, et al. Medical cannabis under statutory health insurance in Germany: Analyses of routine data from 2017 to 2020. BMC Health Services Research. 2022;22(1):122. 10.1186/s12913-022-07543-3.

[13] German Federal Ministry of Health. ECaLe Technical Report: Effects of Cannabis Legalisation – Final Report. Berlin: BMG; July 2023. Available from: https://www.bundesgesundheitsministerium.de/fileadmin/Dateien/5_Publikationen/Drogen_und_Sucht/Abschlussbericht/ecale_technical_report_230721_en_bf.pdf.

[14] Office of Medicinal Cannabis. Medical cannabis programme Netherlands. Ministry of Health, Welfare and Sport. 2024. Available from: https://english.cannabisbureau.nl/.

[15] Israel Ministry of Health. Medical cannabis statistics and regulations. 2024. Available from: https://www.health.gov.il/English/Topics/cannabis/Pages/default.aspx.

[16] Kopeckova B, Mayorga ET, Bass C, et al. Cost-effectiveness of medicinal cannabis for management of refractory symptoms associated with chronic conditions: A systematic review of economic evaluations. PharmacoEconomics - Open. 2021;5(4):613–31. 10.1007/s41669-021-00288-4. 34593176 10.1016/j.jval.2021.04.1276

[17] Whiting PF, Wolff RF, Deshpande S, Di Nisio M, Duffy S, Hernandez AV, et al. Cannabinoids for medical use: a systematic review and meta-analysis. JAMA. 2015;313(24):2456–73. 10.1001/jama.2015.6358. 26103030 10.1001/jama.2015.6358

[18] Lintzeris N, Mills L, Dunlop A, et al. Medical cannabis use in australia: consumer experiences from the online cannabis as medicine survey 2020 (CAMS-20). Harm Reduct J. 2022;19:83. 10.1186/s12954-022-00666-w. 35907959 10.1186/s12954-022-00666-w PMC9338505

[19] Research and Innovation, Centre for Medicinal Cannabis, NSW Government. 2024. Available from: https://www.medicinalcannabis.nsw.gov.au/about/research-and-evidence.

[20] Lambert Initiative for Cannabinoid Therapeutics. Clinical research challenges. University of Sydney. 2023. Available from: https://www.sydney.edu.au/lambert/.

[21] Therapeutic Goods Administration. Medicinal cannabis: access pathways and usage data. December 2024. Available from: https://www.tga.gov.au/products/unapproved-therapeutic-goods/medicinal-cannabis-hub/medicinal-cannabis-access-pathways-and-usage-data.

[22] Paterson P, Nutt C, Isenmann S, et al. The rise and rise of medicinal cannabis, what now? Medicinal cannabis prescribing in Australia 2017–2022. Intern Med J. 2022;52(8):1306–16. 10.1111/imj.15895. 10.3390/ijerph19169853 PMC9408026 36011488

[23] Office of Drug Control. Medical cannabis statistics 2019–2021. Australian Government Department of Health. 2022. Available from: https://www.odc.gov.au/medicinal-cannabis.

[24] Cannabiz. Two-month total of SAS-B approvals up 47% on 2024, TGA data shows. Cannabiz Media; 2025. Available from: https://www.cannabiz.com.au/two-month-total-of-sas-b-approvals-up-47-on-2024-tga-data-shows/.

[25] Lintzeris N, Mills L, Arkell TR, et al. Medical cannabis use in Australia seven years after legalisation: findings from the online cannabis as medicine survey 2022–2023 (CAMS-22). Harm Reduct J. 2024;21:76. 10.1186/s12954-024-00992-1. 38807133 10.1186/s12954-024-00992-1 PMC11131250

[26] Therapeutic Goods Administration. Medicinal cannabis Authorised Prescriber Scheme data. February 2025. Available from: https://www.tga.gov.au/products/unapproved-therapeutic-goods/medicinal-cannabis-hub/medicinal-cannabis-access-pathways-and-usage-data/medicinal-cannabis-authorised-prescriber-scheme-data.

[27] Nasdaq. A state-by-state guide to cannabis in Australia (Updated 2024). 2024. Available from: https://www.nasdaq.com/articles/state-state-guide-cannabis-australia-updated-2024.

[28] Sarris J, Sinclair J, Karamacoska D, et al. Exploring access to medicinal cannabis through general practitioners in Australia. Med J Aust. 2024. 10.5694/mja2.52553.

[29] Cannabis Place. How much does medical cannabis cost in Australia? 2024. Available from: https://www.cannabisplace.com.au/amp/learn/price-medical-marijuana/.

[30] CannaAid Medical. How much does medical cannabis (marijuana) cost in Australia? 2024. Available from: https://cannaaidmedical.com.au/how-much-does-medical-cannabis-cost-in-australia/.

[31] Australian Government Department of Health. PBS co-payment freeze. 2025. Available from: https://www.health.gov.au/cheaper-medicines/pbs-co-payment-freeze.

[32] Grand View Research. Australia medical cannabis market industry report. 2030. 2024. Available from: https://www.grandviewresearch.com/industry-analysis/australia-medical-cannabis-market-report.

[33] Victoria State Government Department of Health. Medicinal cannabis regulatory framework. 2024. Available from: https://www.health.vic.gov.au/drugs-and-poisons/medicinal-cannabis-regulatory-framework.

[34] Therapeutic Goods Administration. Guidance for the use of medicinal cannabis in Australia: Patient information. 2024. Available from: https://www.tga.gov.au/resources/resource/reference-material/guidance-use-medicinal-cannabis-australia-patient-information.

[35] Mills L, Estes L, Schlag AK, et al. Healthcare practitioner perceptions on barriers impacting cannabis prescribing practices. Front Psychiatry. 2022;13:848904. 10.3389/fpsyt.2022.848904. 10.1186/s12906-022-03716-9 PMC9453734 36076191

[36] Therapeutic Goods Administration. GMP Clearance Guidance. Version 18.4. Nov 2023. Available from: https://www.tga.gov.au/sites/default/files/2023-12/gmp-clearance-guidance.pdf.

[37] Therapeutic Goods Administration. Review into the 2016 medicinal cannabis amendments to the Narcotic Drugs Act 1967. 2024. Available from: https://www.tga.gov.au/resources/publication/publications/tabling-report-review-2016-medicinal-cannabis-amendments-narcotic-drugs-act-1967.

[38] Cannabis Law Report. APHRA publishes 2023–24 annual report – medical cannabis telehealth. a concern. 2024. Available from: https://cannabislaw.report/aphra-publishes-2023-24-annual-report-medical-cannabis-telehealth-a-concern/.

[39] Medical Republic. Expect changes in medicinal cannabis regulation. 2025. Available from: https://www.medicalrepublic.com.au/expect-changes-in-medicinal-cannabis-regulation/117030.

[40] PharmOut. Number of medicinal cannabis prescriptions and prescribers in Australia. 2024. Available from: https://www.pharmout.net/number-of-medicinal-cannabis-prescriptions-and-prescribers-in-australia/.

[41] Ahpra & National Boards. Regulating new and changing healthcare. 2025. Available from: https://www.ahpra.gov.au/Resources/Regulating-new-and-changing-healthcare.aspx.

[42] Ahpra & National Boards. Guidance on medicinal cannabis prescribing targets unsafe practice. 2025. Available from: https://www.ahpra.gov.au/sitecore/content/Medical/News/2025-07-09-Medicinal-cannabis-guidance.aspx?utm_source=chatgpt.com.

[43] Little Green Pharma. How much does medical cannabis cost? 2024. Available from: https://www.littlegreenpharma.com/au/pharmacists/costs/.

[44] Tait RJ, Caldicott D, Mountain D, et al. From growers to patients: Multi-stakeholder views on the use of, and access to medicinal cannabis in Australia. PLoS ONE. 2022;17(11):e0277355. 10.1371/journal.pone.0277355. 36367871 10.1371/journal.pone.0277355 PMC9651563

[45] OpenAustralia. Community Affairs References Committee: 16 Mar 2021: Senate debates. 2021. Available from: https://www.openaustralia.org.au/senate/?id=2021-03-16.154.1.

[46] Health Direct. Medicinal cannabis, Australian Government Department of Health. 2024. Available from: https://www.healthdirect.gov.au/medicinal-cannabis.

[47] Arnold JC, Nation T, McGregor IS. Prescribing medicinal cannabis. Aust Prescr. 2020;43:152–9. 10.18773/austprescr.2020.052. 33093741 10.18773/austprescr.2020.052 PMC7572192

[48] Queensland Health. Prescribing medicinal cannabis in Queensland. 2024. Available from: https://www.health.qld.gov.au/public-health/topics/medicinal-cannabis/prescribing.

[49] New South Wales Parliament. Inquiry into the current barriers to patient access to medicinal cannabis in NSW. 2024. Available from: https://www.parliament.nsw.gov.au/committees/inquiries/Pages/inquiry-details.aspx?pk=2856.

[50] NSW Health. Prescriptions, dispensing, storage and record keeping of cannabis medicines. 2024. Available from: https://www.health.nsw.gov.au/pharmaceutical/cannabismedicines/Pages/prescriptions-and-dispensing.aspx.

[51] Patient advocacy submissions to Senate inquiry on barriers to medicinal cannabis access. 2024. Available from: https://www.aph.gov.au/Parliamentary_Business/Committees/Senate/Community_Affairs/MedicinalCannabis/Submissions.

[52] Therapeutic Goods Administration. Planned consultation to address growing safety concerns of unapproved medicinal cannabis products in Australia. 2025. Available from: https://www.tga.gov.au/news/news/planned-consultation-address-growing-safety-concerns-unapproved-medicinal-cannabis-products-australia#:~:text=This%20consultation%2C%20which%20will%20commence%20on%2011,those%20containing%20higher%20levels%20of%20tetrahydrocannabinol%20(THC).

